# Combining CSPG4-CAR and CD20-CCR for treatment of metastatic melanoma

**DOI:** 10.3389/fimmu.2023.1178060

**Published:** 2023-10-11

**Authors:** Karin Teppert, Nora Winter, Vera Herbel, Caroline Brandes, Simon Lennartz, Fabian Engert, Andrew Kaiser, Thomas Schaser, Dominik Lock

**Affiliations:** Miltenyi Biotec B.V. & Co. KG, Bergisch Gladbach, Germany

**Keywords:** immunotherapy, adoptive T cell therapy, chimeric antigen receptor, chimeric costimulatory receptor, melanoma

## Abstract

The prognosis for patients with metastatic melanoma is poor and treatment options are limited. Genetically-engineered T cell therapy targeting chondroitin sulfate proteoglycan 4 (CSPG4), however, represents a promising treatment option, especially as both primary melanoma cells as well as metastases uniformly express CSPG4. Aiming to prevent off-tumor toxicity while maintaining a high cytolytic potential, we combined a chimeric co-stimulatory receptor (CCR) and a CSPG4-directed second-generation chimeric antigen receptor (CAR) with moderate potency. CCRs are artificial receptors similar to CARs, but lacking the CD3ζ activation element. Thus, T cells expressing solely a CCR, do not induce any cytolytic activity upon target cell binding, but are capable of boosting the CAR T cell response when both CAR and CCR engage their target antigens simultaneously. Here we demonstrate that co-expression of a CCR can significantly enhance the anti-tumor response of CSPG4-CAR T cells *in vitro* as well as *in vivo*. Importantly, this boosting effect was not dependent on co-expression of both CCR- and CAR-target on the very same tumor cell, but was also achieved upon trans activation. Finally, our data support the idea of using a CCR as a powerful tool to enhance the cytolytic potential of CAR T cells, which might open a novel therapeutic window for the treatment of metastatic melanoma.

## Introduction

Great clinical success has been achieved with the implementation of CAR T cells, especially for treatment of hematological malignancies (1–3). However, liquid tumors only represent 8-10% of all adult human cancers and certain characteristics of solid tumors such as strong physical barriers inhibiting T cell infiltration and a highly immunosuppressive tumor microenvironment have been demonstrated to weaken adoptive T cell therapies (4–6). In addition, one major challenge represents the selection of a suitable tumor antigen to be targeted, since most of the identified tumor-associated antigens are also expressed on healthy tissues, carrying the risk for on-target/off-tumor toxicity and severe side effects (7). This is for instance the case for the target antigen CSPG4, first identified in melanoma and thus also referred to as melanoma-associated-chondroitin-sulfate-proteoglycan (MCSP) (8). Although expression levels are clearly lower than in tumor cells, CSPG4 is also found on non-malignant tissue, such as pericytes and small intestine (9, 10). Nonetheless, CSPG4 represents a promising target for adoptive T cell therapies, as it is not only overexpressed in melanoma but also in a broad range of other malignancies such as triple-negative breast cancers, various types of gliomas, head and neck squamous-cell carcinomas, soft-tissue sarcomas, tumor-associated vasculature and also leukemia (11–13). In line with this, CSPG4 was described to promote multiple steps of cancer development such as angiogenesis, dissemination, metastasis, proliferation, and survival (14, 15). The expression in not only primary but also metastatic melanoma cells further underlines the great therapeutic potential of targeting CSPG4, especially for treatment of metastatic melanoma, which is generally associated with poor prognosis and a median survival of less than one year (9, 10, 16). Traditional approaches such as chemotherapy, radiotherapy and surgical removal are ineffective in late-stage metastasized melanoma (17). Targeted immunotherapies such as MEK- or BRAF-inhibitors have led to promising results, but the high mutational rate in melanoma strongly promotes the development of secondary resistances (18). The implementation of immune checkpoint blockade (ICB) has yielded a groundbreaking outcome, marking the first successful extension of survival for metastatic melanoma (19, 20). However, latest reports from clinical trials (NCT01844505) showed that long-lasting effects can only be expected in approximately half of the patients (21, 22). Adoptive T-cell based immunotherapies targeting for instance VEGFR-2 (NCT01218867), GD2 (NCT02107963, NCT03635632), CD70 (NCT02830724), gp100 (NCT03649529) or CD20 (NCT03893019) are currently assessed in several clinical trials and were suggested to be especially helpful in case of treatment-resistant melanomas (23). Particularly combinatorial approaches were suggested to increase efficiency and to minimize the risk for therapy-accompanied adverse events such as on-target/off-tumor toxicity (23). This aspect of increasing tumor cell specificity is also highly relevant in the context of targeting CSPG4, due to its role in multiple physiological processes and the expression on healthy tissues (24). To this end, our approach was focused on combining a low-affinity CSPG4-CAR, which by itself only showed weak activity and cytotoxicity, with a chimeric co-stimulatory co-receptor (CCR). CCRs are artificial receptors, which comprise an extracellular binding moiety, a spacer, a transmembrane region and defined intracellular signaling domains that differ from conventional CAR designs. As the CD3ζ signaling domain is typically absent in CCRs, no cytolytic activity is induced upon antigen engagement. However, CCRs are capable of boosting a simultaneously activated CAR T cell response, which results in an enhanced release of inflammatory cytokines and increased cytotoxicity. Due to the low functional avidity, the risk for CAR-binding to healthy target cells expressing low levels of CSPG4 is reduced. We show that CSPG4-expressing target cells were only effectively lysed when both CCR and CAR engaged their target antigens. This means that the CCR could either simply target a second tumor-associated antigen, or an antigen, which is not expressed by the tumor cell but found in close proximity, as for example in the tumor microenvironment (TME). In this study, CD20 was selected as CCR target; on the one hand to guarantee omnipresent expression in B cells, and on the other hand, due to the clinical success by targeting CD20^+^ cell subsets in melanoma (9, 10, 24–26).

## Results

### Generation of a panel of CCR variants expressing different co-stimulatory domains

**Figure 1:**
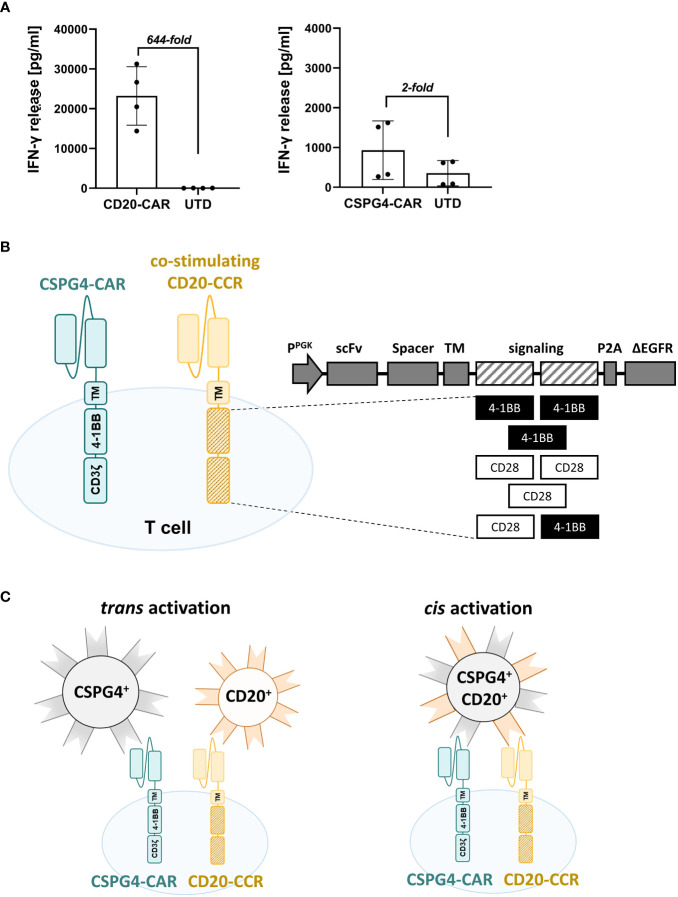
Co-expressing CD20-targeting Chimeric Co-stimulation Receptor (CCR) to boost CSPG4 CAR functionality. **(A)** Fold-increase in IFN-γ release by high-performing CD20-CAR T cells (right graph) in comparison to low-affinity antibody-derived CSPG4-CAR T cells (left graph) upon 24 hours co-culture with CD20^+^ JeKo-1 cells or CSPG4^+^ Mel526 cells, respectively. Data shows mean and individual values for four different donors (± SD). **(B)** Schematic representation of a T cell co-expressing a 4-1BB-co-stimulated second-generation CSPG4-CAR and a chimeric CD20-CCR, containing one or two co-stimulatory domains (4-1BB_4-1BB; 4-1BB, CD28_CD28; CD28; CD28_4-1BB) and lacking the CD3ζ signaling domain. **(C)** Mechanism of boosting the performance of the low-performing CSPG4-CAR (blue) by co-expression of a CD20-CCR (yellow), either achieved through cis (left) or trans (right) activation, meaning that CCR- and CAR- target are expressed on the same target cell or on different target cells, respectively.

With the primary goal to improve the safety of CSPG4-targeting CAR T cell therapies, we used a low-affinity antibody-derived CSPG4-CAR, which showed low cytotoxic activity, consequently entailing a minimized risk for on-target/off-tumor toxicity. As expected, comparison of CAR T cells, either expressing the conventional Leu16-derived CD20-CAR or a CSPG4-CAR, revealed more than 20-fold lower levels of IFN-γ release with the novel low-affinity CSPG4-CAR (**Figure 1A**). Aiming to boost the activity of this CSPG4-CAR, various CD20-targeting CCR constructs with different combinations of 4-1BB and/or CD28 co-stimulatory domains were generated (**Figure 1B**). CD20 was selected as CCR target in order to guarantee omnipresent expression through bystanding or tumor-infiltrating B cells (9, 10, 24–26). The hypothesized mechanism is illustrated in **Figure 1C**, showing that target cell lysis is only successfully facilitated by CSPG4-CAR T cells, when both CAR and the co-expressed CCR engage their target antigens simultaneously. As depicted, CAR- and CCR-target can either be expressed in cis (on the same target cell) or in trans (on two different target cells).

### CCRs boost the cytolytic potential of CSPG4-directed CAR T cells

**Figure 2:**
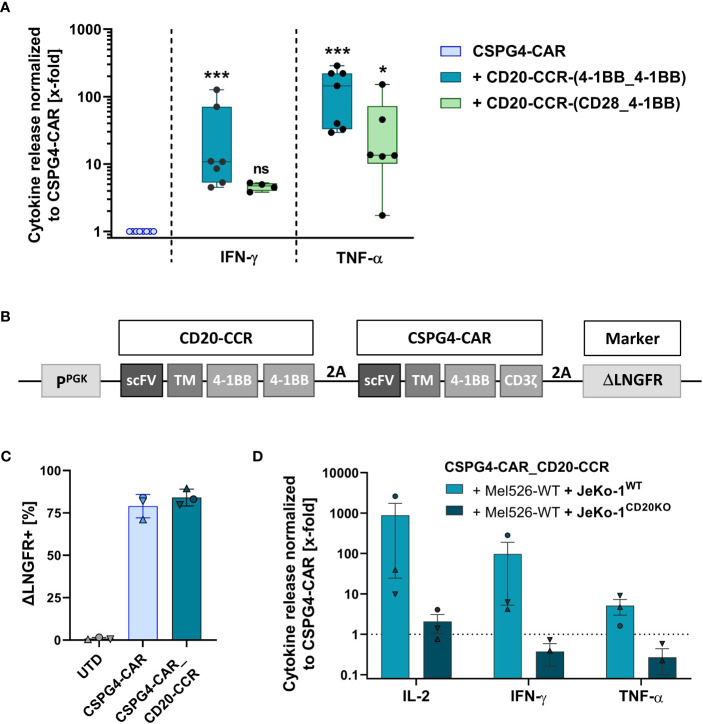
Co-expression of CSPG4-CAR and CD20-CCR significantly increases cytokine release upon trans activation and can be achieved through transduction with a polycistronic lentiviral construct. **(A)** T cells were lentivirally transduced with a CSPG4-CAR and/or a CD20-CCR, either containing 4-1BB_4-1BB or CD28_4-1BB costimulatory domains. Cytokine secretion of IFN-γ and TNF-α was determined after 24 hours of co-culture with CSPG4^+^ Mel526 and CD20^+^ JeKo-1^WT^ cells. Box and whiskers plots show median and individual values (normalized to CSPG4-CAR) for eight different donors from four individual experiments. Significance was determined using nonparametric one-way ANOVA (ns, not significant; *ρ ≤ 0.05, ***ρ ≤ 0.001). **(B)** Gene schematics of CD20-CCR_CSPG4-CAR T cells, poly-cistronically expressing CSPG4-CAR and CD20-specific CCR-(4-1BB_4-1BB). **(C)** Frequency of magnetically enriched ΔLNGFR^+^, measured before co-culture with target cells. Mean and individual values are shown for four different donors from two independent experiments (± SD). **(D)** Cytokine secretion (IL-2, IFN-γ, TNF-α) after 24 hours of co-culture with a mixture of CSPG4^+^ Mel526 and either CD20^+^ JeKo-1^WT^ cells or CD20^-^ JeKo-1^CD20KO^ cells at an E:T ratio of 5:1. Bar graph shows mean and individual values for three different donors (± SEM).

In order to test whether a CCR boosts the cytolytic potential of the CSPG4-CAR, activated T cells were co-transduced with the CSPG4-CAR and with one of the engineered CD20-CCR variants. Prior to functionality analysis, the T cells were enriched via the surface markers ΔLNGFR and/or ΔEGFR (exemplarily shown in **Figure S1A**). To initially study the approach in trans and to assess whether the boosting effect is dependent on CCR engagement with its target antigen, T cells co-expressing the CSPG4-CAR and a specific CCR construct were co-cultured with CSPG4^+^ Mel526 cells *in vitro*, in presence or absence of CD20^+^ JeKo-1^WT^ cells. After 24 hours, the level of pro-inflammatory cytokines in the supernatant was determined. Since strong 4-1BB signaling supports survival and persistence of CAR T cells, we focused on two different CCR constructs with either two sequential 4-1BB endodomains or the combination of 4-1BB and CD28 co-stimulatory domain, termed CCR-(4-1BB_4-1BB) and CCR-(CD28_4-1BB), respectively. Strongest effect upon dual stimulation was observed with CSPG4-CAR T cells co-expressing CCR-(4-1BB_4-1BB), resulting in significantly increased IFN-γ and TNF-α production compared to CSPG4-CAR T cells only (**Figure 2A**). In general, all CCR variants led to increase in cytokine secretion with more than 10- and 100-fold higher IFN-γ and TNF-α release, respectively, whereas control groups with T cells expressing only CAR or only CCR did not show noticeable release of pro-inflammatory cytokines (exemplarily shown in **Figures S1B, C**). The data demonstrated that the functionality of the CSPG4-CAR was significantly increased through co-expression of a CCR and, as previously hypothesized, that the combination of CAR and CCR only facilitated increased potency when both CAR and CCR were engaging their target antigens.

In order to simplify the engineering process, to ensure purity of the final T cell product (containing neither CAR-only nor CCR-only T cells), we designed a polycistronic lentiviral construct, encoding for both CSPG4-CAR and CD20-directed CCR-(4-1BB_4-BB), termed CSPG4-CAR_CD20-CCR (**Figure 2B**). CCR and CAR co-expressing T cells were engineered using the newly designed polycistronic lentiviral vector and subsequently enriched via the transduction marker ΔLNGFR, reaching comparable transgene expression above 75% (**Figure 2C**). After 24 hours of co-culture in trans, CSPG4-CAR_CD20-CCR T cells demonstrated strongly enhanced secretion of IL-2, IFN-γ and TNF-α, compared to CSPG4-CAR T cells only (**Figure 2D**). As expected, this boosting effect was dependent on the presence of CD20^+^ JeKo-1^WT^ cells. Co-culture with a mixture of Mel526^WT^ and JeKo-1^CD20KO^ cells led to comparable cytokine release between CSPG4-CAR T cells and dual-specific CSPG4-CAR_CD20-CCR cells.

**Figure 3:**
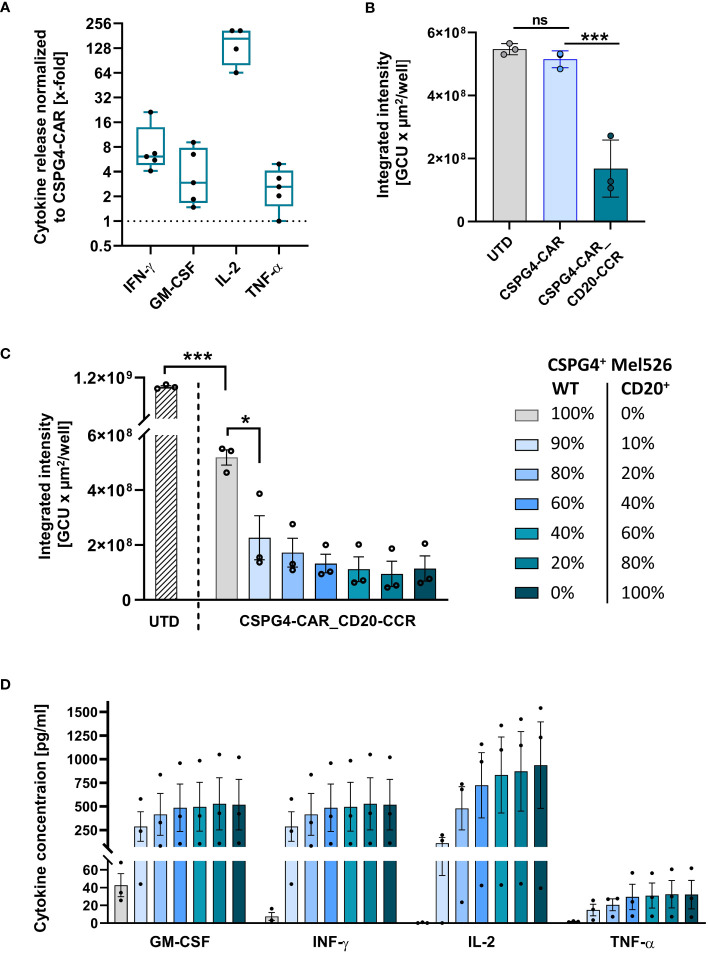
CSPG4-CAR_CD20-CCR T cells demonstrate significantly enhanced cytotoxicity upon cis stimulation and respond in a dose-dependent manner. **(A)** Cytokine release by CD20-CCR_CSPG4-CAR T cells after 24 hours of co-culture with CD20^+^ CSPG4^+^ Mel526^CD20^ target cells an E:T ratio of 1:1. Box and whiskers plots show median and individual x-fold values, normalized to CSPG4-CAR T cells, for five different donors from two individual experiments. **(B)** Integrated intensity of GFP^+^ CD20-CCR_CSPG4-CAR T cells after 94 hours co-culture with UTD (untransduced) T cells, CSPG4-CAR T cells, or CD20-CCR_CSPG4-CAR T cells at an E:T ratio of 1:1. Displayed are mean and individual values (± SD). Significance was determined using ordinary one-way ANOVA (ns, not significant; *ρ ≤ 0.05, ***ρ ≤ 0.001). **(C)** UTD T cells and CD20-CCR_CSPG4-CAR T cells were co-cultured for 94 hours with varying percentages of Mel526^WT^ and Mel526^CD20^ target cells at an E:T ratio of 2:1. Displayed is the integrated intensity of GFP^+^ target cells, showing mean and individual values of three different donors (± SEM). Significance was determined using ordinary one-way ANOVA (*ρ ≤ 0.05, ***ρ ≤ 0.001). **(D)** Cytokine concentrations in the supernatant were determined after 24 hours.

After proving the applicability of the boosting system in the trans setting, we next aimed to assess, whether cytokine secretion of CSPG4-CAR_CD20-CCR T cells is also enhanced upon cis activation, using CD20^+^ CSPG4^+^ Mel526 cells. In accordance with the results obtained in trans, CSPG4-CAR_CD20-CCR T cells led to significantly increased, more than 5-fold higher, IFN-γ and GM-CSF secretion than CSPG4-CAR T cells without CCR (**Figure 3A**). Particularly, the IL-2 secretion was strongly increased in CSPG4-CAR_CD20-CCR T cells, demonstrating more than 100-fold higher production than CSPG4-CAR T cells. Moreover, co-expression of CCR-(4-1BB_4-1BB) significantly enhanced the *in vitro* cytolytic potential and recovered functionality of the CSPG4-CAR (**Figure 3B**). Lysis of CD20^+^ CSPG4^+^ Mel526 target cells was comparable between untransduced T cells and CSPG4-CAR T cells, whereas only dual-stimulated CSPG4-CAR_CD20-CCR T cells demonstrated significant cytotoxicity.

### CCR boosts CAR in a dose-dependent manner

As hypothesized, we were able to show that the cytotoxic potential of our low-affinity antibody-derived CAR, which on its own only showed moderate activity and consequently also minimized risk for on-target/off-tumor toxicity, can be recovered in a target-specific manner by co-expression of the CD20-targeting CCR-(4-1BB_4-1BB). To assess whether this boosting effect through the CCR in this artificial system is dependent on the CCR-target antigen level, we co-cultured CSPG4-CAR_CD20-CCR T cells with CSPG4^+^ Mel526^WT^ target cells and spiked in defined percentages of CD20-expressing CSPG4^+^ Mel526^CD20^ cells. This titration experiment, which is especially relevant in case of reduced CCR-target antigen availability, revealed that only 10% of CCR antigen expressing target cells were already sufficient to significantly induce lysis of CSPG4^+^ target cells with CSPG4-CAR_CD20-CCR T cells in a dose-dependent manner. The higher the frequency of CD20^+^ cells, the stronger the CAR-induced cytotoxicity and clearance of Mel526 cells (**Figure 3C**). Consistently, engagement of CD20 via the CCR was required for full cytokine secretion, shown by the dose-dependent increase in GM-CSF, IFN-γ, IL-2 and TNF-α in the supernatant after 24 hours of co-culture (**Figure 3D**).

Considering this relatively low CCR-activation threshold and the potential on-target/off-target toxicity against CSPG4^low^-expressing healthy cells through CD20^+^ bystander cells, we performed a side-by-side comparison of CSPG4-CAR_CD20-CCR T cells co-cultured with either CSPG4^high^ or CSPG4^low^ target cells (exemplarily shown in **Figures S2A–C**). As hypothesized, we observed that co-culture with CSPG4^low^ A-431 cells (in presence of CCR-stimulating JeKo-1^WT^ cells) did not induce proliferation nor increase cytotoxicity of CSPG4-CAR_CD20-CCR T cells.

### CCR boosts CAR *in vivo* and mediates robust tumor regression in a melanoma model

**Figure 4:**
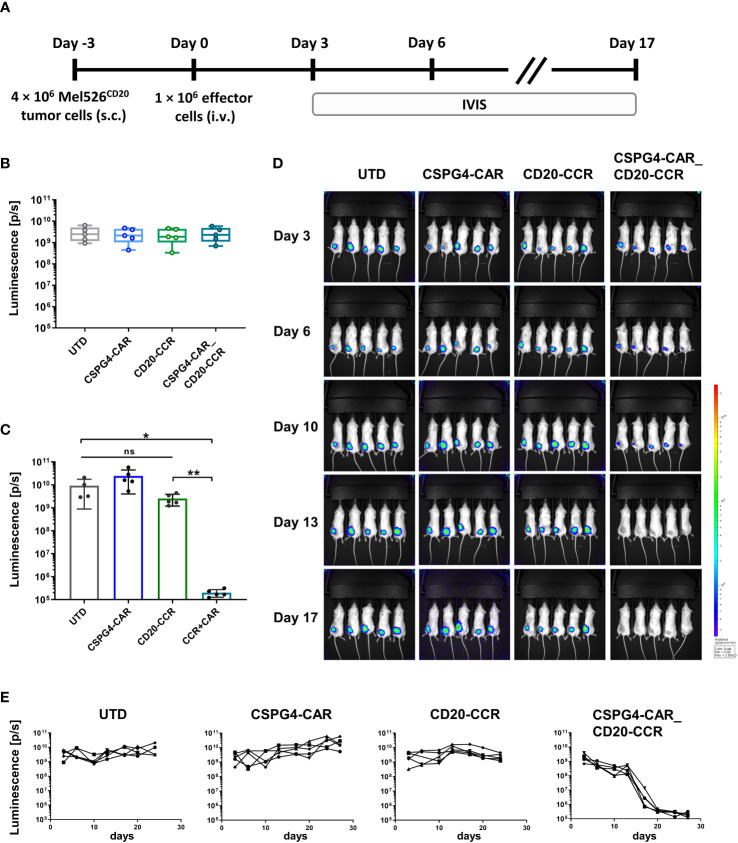
*In vivo* tumor clearance only successfully achieved upon treatment with CSPG4-CAR_CD20-CCR T cells. **(A)** Experimental timeline comparing antitumor efficacy of CSPG4-CAR, CD20-CCR and CSPG4-CAR_CD20-CCR in mice bearing Mel526FFluc_CD20_eGFP cells. **(B)** Randomization of different treatment groups with 5 mice each. **(C)** Endpoint-analysis of the anti-tumor reactivity of UTD, CSPG4-CAR, CD20-CCR or CSPG4-CAR_CD20-CCR was assessed using *in vivo* imaging systems (IVIS) measurement. **(D)** IVIS measurements displaying tumor burden on day 3, 6, 10, 13 and 17. **(E)** Luminescence measured over the course of the whole study displayed for mouse treated with UTD T cells, CSPG4-CAR T cells, CD20-CCR T cells and CSPG4-CAR_CD20-CCR co-expressing T cells. Significance was determined using nonparametric Mann-Whitney t-test (ns = not significant, *ρ ≤ 0.05, **ρ ≤ 0.01).

In order to study the cytolytic potential of CSPG4-CAR_CD20-CCR T cells *in vivo* with solid tumors, a melanoma xenograft model was established with CD20^+^ Mel526 tumor cells (**Figure 4A**). After tumor engraftment, mice were randomized according to tumor size. Subsequently, 1 × 10^6^ UTD T cells, CSPG4-CAR T cells, CD20-CCR T cells, or CSPG4-CAR_CD20-CCR T cells were injected intravenously (**Figure 4B**). In line with the *in vitro* findings, CSPG4 CAR T cells led to outgrowth of tumor cells, while tumor clearance was only observed in the group with CCR-expressing CSPG4-CAR T cells (**Figures 4C–E**). Mice treated with CSPG4-CAR T cells showed continuous outgrowth of the engrafted melanoma cells, similar as observed for groups treated with UTD or CCR-only T cells. In summary, this study showed that the moderate cytotoxic potential of the low-affinity antibody-derived CSPG4-CAR could successfully be recovered *in vitro* as well as *in vivo* by co-expression of a 4-1BB_4-1BB-costimulated CD20-CCR.

## Discussion

The rather poor prognosis for patients with metastatic malignant melanoma is associated with a median survival of less than one year (16, 27). CSPG4-targeting adoptive T cell therapy, however, might represent a powerful treatment option, especially as CSPG4 is not only expressed in primary but also metastatic melanoma cells (11, 13). Moreover, CSPG4 was also found in a broad range of other malignancies such as triple-negative breast cancer, various types of gliomas, head and neck squamous cell carcinoma, soft-tissue sarcomas, tumor-associated vasculature and also leukemia (9, 10). This is in line with the reported role of CSPG4 in several cancer-associated pathways, including angiogenesis, dissemination, metastasis, proliferation and survival (13, 14). It is important to keep in mind that low levels of CSPG4 were also found in non-malignant tissue, which might cause severe side effects due to on-target/off-tumor toxicity (28–30). For this reason, our approach was focused on combining a low-affinity CSPG4-targeting CAR, which by itself only showed weak anti-tumor cytotoxicity, with an anti-CD20 chimeric co-stimulatory co-receptor (CCR). As hypothesized, CSPG4-expressing tumor cells were only efficiently lysed when both CCR and CAR engaged their target antigens simultaneously. Interestingly, boosting of the CAR T cell response was not only observed upon cis activation (both targets expressed on same tumor cell) but also in case of trans activation (targets on two different tumor cells). This is a very promising finding for clinical application as targeting of CSPG4^+^ primary and metastatic melanoma cells can be facilitated via trans stimulation through abundantly available bystanding or even tumor-infiltrating CD20-expressing B cells (31). Additionally, boosting through cis activation allows the targeting of tumor-initiating CD20^+^ CSPG4^+^ melanoma cancer stem cells, which is supported by the clinical success using the anti-CD20 antibody Rituximab for treatment of melanoma (25, 26, 32–34).

We demonstrated that the co-expression of a CD20-CCR, encoding two sequential 4-1BB co-stimulatory domains, led to significantly increased target cell-specific cytotoxicity of the CSPG4-CAR *in vitro* and *in vivo*. Although the superiority of this CCR was not very pronounced, aiming to ensure survival and persistency of our gene-engineered T cells, we specifically focused on a strong 4-1BB signaling which is known – in contrast to CD28 signaling - to specifically upregulate transcription factors associated with memory differentiation and anti-apoptotic pathways (35). The melanoma xenograft model with CD20^+^ Mel526 tumor cells clearly demonstrated that treatment with CSPG4-CAR T cells led to continuous tumor outgrowth, while robust tumor regression was only achieved upon treatment with dual-specific CSPG4-CAR_CD20-CCR T cells. This again substantiates the need for simultaneous target antigen engagement through both CAR and CCR, which highly increases the safety and tumor cell restriction of CSPG4-targeted T cell therapy. Aiming for the same goal of reducing the risk for on-target/off-tumor toxicity, Kloss *et al.* showed that also a first-generation CD19-CAR can be functionally rescued through combination with a PSMA-targeting CCR (36). While validating this finding for the target antigen CSPG4, we were also able to successfully extend the understanding by analyzing multiple CCR molecules with various co-stimulatory domains, the influence of the CCR target antigen level, and the applicability of this combinatorial approach in cis and in trans. In this context, the finding that even low frequencies of CCR antigen-expressing target cells were sufficient to significantly enhance the cytolytic capacity is of particular importance for potential clinical applications, where the CCR target might be the limiting factor. Further studies, though, are required to assess whether this low CCR-activation threshold – despite the combination with low-affinity antibody-derived CARs - would then also increase the risk for on-target/off-tumor toxicity. In-depth validation is required to assess the extent of bystander lysis of CD20^+^ cells. However, since the CD20-targeting CCR by itself was shown to be non-functional, only minor depletion of non-malignant bystander B cells is expected, while the side effects should be clinically tolerable, especially considering the success of leukemia or lymphoma treatment with CD20- or CD19-targeting CAR T cells (37).

A clinical study using Her2-targeting CAR T cells drastically demonstrated the necessity to increase safety of CAR T cell therapies, as already low levels of target antigen expression on healthy tissue led to on-target/off-tumor toxicity causing lethal adverse effects (7). Novel technologies such as the adCAR, UniCAR, or inducible CAR allow for temporal “on-/off-switching” and a controllable CAR T cell response (28–30). However, all of those systems require regular re-injection of the respective stimulus and the clinical long-term efficacy still needs to be evaluated. Wiesinger *et al.* used mRNA electroporation in order to achieve transient expression of the CSPG4-CAR and to minimize the risk for side effects in the clinical trial (38). Again, this approach necessitates complete tumor clearance and might have insufficient long-term effect to prevent recurrence. Another approach for safer adoptive cell therapy is based on Boolean logic AND gates, in which an affinity-reduced first-generation CAR containing only the CD3ζ activation signal is combined with a CCR providing the co-stimulatory domain (39). Similar to this, it was also our goal to achieve a perfectly calibrated and balanced split antigen recognition in order to only induce full T cell response, when both CAR and CCR are stimulated simultaneously. However, our CAR is not necessarily dependent on CCR stimulation and shows functionality by itself. Despite of the very low potency of our CAR, this might still represent a safety problem, which needs to be clinically evaluated. The use of a second-generation CAR with reduced potency offers the advantage of minimizing the risk of antigen escape, whereas target cell lysis is only expected in malignant tissues with sufficient CSPG4 expression levels to induce CAR-T cell activation.

In regard to clinical applicability, we also demonstrated that T cells expressing CAR and CCR can be successfully manufactured by using only one polycistronic lentiviral vector, even showing slightly higher anti-tumor activity compared to co-transduction. In order to apply our approach to other clinically relevant settings, it would also be interesting to study the functionality of CAR T cells co-expressing a CCR, targeting the tumor microenvironment. Especially in the context of solid tumors, various studies demonstrated the beneficial effect of CARs targeting components of the TME such as TGF-β (15) or fibroblast activating protein (40–42). Moreover, the efficacy and functional persistence of CSPG4-CAR_CD20-CCR T cells could further be amplified through combination with ICB, especially considering its great clinical progress in treatment of advanced melanoma (43) and the encouraging results of combining ICB and adoptive cell therapy observed in preclinical and clinical studies (44–47).

Finally, our *in vitro* and *in vivo* findings confirm that co-expression of a CD20-directed CCR successfully potentiated the anti-tumor cytotoxicity of CSPG4-CAR T cells in a CCR- and CAR- target antigen-dependent manner. In light of the fact that this boosting effect was achieved upon cis and trans activation, this approach opens a novel therapeutic window by targeting not only primary and metastatic tumor cells but also tumor-promoting melanoma cancer stem cells (25, 26, 32–34, 48). However, this combinatorial adoptive T cell-based approach might be of particular relevance in case of advanced late-stage melanomas, when traditional therapy such as chemotherapy, radiotherapy or surgical removal are ineffective (17, 23).

## Conclusion

We showed that our CD20-targeting CCRs enhance the cytolytic potential and polyfunctionality of the co-expressed CSGP4-CAR, not only upon a simultaneous activation but also in a CCR-target antigen-dependent manner. Depending on target antigen expression, safety and toxicity might vary for different CCR and CAR combinations and need to be evaluated individually. However, our data suggests that especially the combination of CCRs with low-affinity antibody-derived CARs, which depend on high-level target antigen expression and consequently spare basal tissues, represents a promising therapeutic concept for the treatment of a wide range of solid tumors. 

## Methods

Unless mentioned to the contrary, kits were used according to the manufacturer´s protocol.

### Generation of engineered cell lines

Mel526 cells (CVCL_8051) were lentivirally transduced to express a PGK promotor-driven construct encoding FFluc_CD20_eGFP. After 72 hours, CD20^+^ cells were enriched using anti-CD20 Biotin (Miltenyi Biotec, #130-113-372) and anti-Biotin-Microbeads (Miltenyi Biotec, #130-105-637) and LS columns (Miltenyi Biotec, #130-122-729). Subsequently, cells were seeded in a limiting dilution for 2 weeks. Individual clones were analyzed using flow cytometry and frozen. Mel526FFluc_CD20_eGFP cells are referred to as Mel526^CD20^.

JeKo-1 CD20 knock-out cells (JeKo-1^CD20KO^) were generated as previously described (49). In brief, 1 µg gRNAs targeting 5´-CACGCAAAGCTTCTTCATGA-3´ (Metabion) were co-transfected with 1 µg Cas9 (Integrated DNA Technologies, Coralville, USA) encoding plasmid using Nucleofector 2b Device and the Cell line Nucleofector Kit V VCA-1003 (Lonza Group, Basel, Switzerland). After seven days, CD20^+^ cells were depleted using LD columns (Miltenyi Biotec, #130-042-901) and anti-CD20 Biotin (Miltenyi Biotec, #130-113-372) and anti-Biotin-Microbeads (Miltenyi Biotec, #130-105-637). Hereafter, 0.3 cells/well were seeded in a 96-well culture plate (Corning, #3598) and a single cell expansion was performed for two weeks. JeKo-1^CD20KO^ cells were analyzed by flow cytometry and frozen.

### Cloning and generation of engineered CSPG4-CAR, CD20-CCR and CSPG4-CAR_CD20-CCR CAR T cells

The CSPG4-specific second-generation CAR sequence encodes a 225.28s-derived scFv linked to an IgG4 Hinge_CH2_CH3 spacer, a 4-1BB co-stimulatory, CD3ζ signaling domain and a P2A-connected ΔLNGFR. The CD20-directed CCR library contained a Leu-16-derived scFv, a spacer domain, different combinations of 4-1BB and CD28 as co-stimulatory domains and P2A-linked ΔEGFR. In the PGK-promotor-driven construct encoding CCR as well as CAR, the CCR-(4-1BB_4-1BB) was P2A element-linked to the CSPG4-CAR followed by a T2A element-linked ΔLNGFR.

T cells were isolated from healthy donor-derived Buffy coats using the PAN T cell isolation Kit (Miltenyi Biotec, #130-096-535). T cells were activated using T Cell TransAct™, human (Miltenyi Biotec, #130-111-160). After 24 hours, T cells were transduced with lentiviral particles and cultured in TexMACS (Miltenyi Biotec, #130-097-196), supplemented with 12.5 ng/ml human IL-7 (Miltenyi Biotec, #130-095-367) and human IL-15 (Miltenyi Biotec, #130-095-760), respectively. CAR and/or CCR (co-)expressing T cells were enriched after 7 days via their co-expressed marker genes ΔLNGFR and ΔEGFR using either anti-ΔLNGFR-Biotin (Miltenyi Biotec, #130-112-797) anti-Biotin MultiSort-MB Kit (Miltenyi Biotec, #130-091-256) or anti-ΔEGFR-PE (Miltenyi Biotec, #130-115-505) and anti-PE-MB (Miltenyi Biotec, #130-105-639), respectively.

### Functionality testing of engineered T cells

Cytolytic activity was assessed by co-culturing either untransduced or modified effector cells with 1 × 10^4^ GFP^+^ target cells. Trans stimulation was facilitated through co-culture with a mixture of Mel526^WT^ and either CD20^+^ JeKo-1^WT^ or CD20^-^ JeKo-1^CD20KO^ cells. Cis stimulation was achieved through co-culture with CD20-transduced Mel526^CD20^ target cells. Effector-to-target ratios and time of co-culture are indicated in the figure legends. All experiments were performed using technical duplicates. Specific killing was calculated based on the number of residual target cells measured by flow cytometry using MACSQuant Analyzer 10 (Miltenyi Biotec, Bergisch Gladbach, Germany) or using the IncuCyte S3 Live-Cell Analysis Systems (Essen BioScience, Michigan, USA), determining the integrated intensity of GFP^+^ target cells in GCU x µm/well. Cytokine concentrations were determined in supernatant after 24 hours of co-culture using the human MACSPlex Cytokine 12 Kit (Miltenyi Biotec, #130-099-169).

All experiments performed on animals follow institutional guidelines and regulations. 6- to 8-week-old female NOD.Cg-PrkdcscidIl2rgtm1Wjl/SzJ NSG mice were purchased from Charles River and kept under specific pathogen-free conditions. Mice were kept at 12:12 light/dark cycles with unrestricted food and water supply. 4 × 10^6^ Mel526^CD20^ cells were injected subcutaneously in the flanks. When a tumor size of 0.5 cm² was reached, mice were randomized into treatment groups, each containing 5 mice. 1 × 10^6^ effector cells were infused intravenously (i.v.). Tumor burden was monitored twice a week using an *in vivo* Imaging System IVIS Lumina III (Perkin Elmer, Waltham, USA) after intraperitoneal D-Luciferin (Gold Biotechnology, #LUCK-1G) injection.

### Statistical analysis

Statistical significance was determined using GraphPad Prism version 8.1.2. The used test is described in the figure legends. The ρ-values are indicated by following criteria: ns, not significant; *ρ ≤ 0.05, **ρ ≤ 0.01, ***ρ ≤ 0.001, ****ρ ≤ 0.0001).

## Data availability statement

The raw data supporting the conclusions of this article will be made available by the authors, without undue reservation.

## Ethics statement

Ethical approval was not required for the studies involving humans because Buffy coats were provided by Klinikum Dortmund with written informed consent before sample collection. The studies were conducted in accordance with the local legislation and institutional requirements. The human samples used in this study were acquired from Buffy coats were provided by Klinikum Dortmund with written informed consent before sample collection. Written informed consent to participate in this study was not required from the participants or the participants’ legal guardians/next of kin in accordance with the national legislation and the institutional requirements. The animal study was approved by Landesamt für Natur, Umwelt und Verbraucherschutz Nordrhein-Westfalen. The study was conducted in accordance with the local legislation and institutional requirements.

## Author contributions

DL and AK conceptualized the study; KT, NW, VH, CB, SL, FE and DL generated and analyzed data; AK supervised defined aspects of the project; KT and DL wrote a first draft of the manuscript. TS reviewed and edited the manuscript. All authors contributed to the article and approved the submitted version.

## Supplementary material

Supplementary Figure 1 Co-expression of CSPG4-CAR and CD20-CCRs, containing various co-stimulatory domains, significantly increases cytokine release upon trans activation. **(A)** T cells were lentivirally transduced with a CSPG4-CAR, CD20-CCRs (different variants with alternative endodomains displayed in parenthesis) or a combination of both CSPG4-CAR and CD20-CCR (blue bars). The level of (co-)expression is shown pre- and post-enrichment. CAR and CCR expression was determined via co-expression of surface markers ΔLNGFR and ΔEGFR, respectively. Exemplarily shown are mean and individual values of two different donors (± SD). **(B, C)** Dual-specific T cells co-expressing CSPG4-CAR and CD20-CCR and T cells only expressing CD20-CCR were co-cultured with CSPG4^+^ Mel526 with our without CD20^+^ JeKo-1^wt^ cells. Displayed is the IFN-γ secretion after 24 hours of co-culture. Significance was determined using ordinary one-way ANOVA (ns, not significant; *ρ ≤ 0.05, **ρ ≤ 0.01, ***ρ ≤ 0.001, ****ρ ≤ 0.0001). Shown are mean and individual values of two different donors (± SD). **(D, E)** Dual-specific T cells co-expressing CSPG4-CAR and CD20-CCR and T cells only expressing CD20-CCR or CSPG4-CAR were co-cultured with CSPG4^+^ Mel526 with our without CD20^+^ JeKo-1^wt^ cells. Displayed is the TNF-α secretion after 24 hours of co-culture. Box and whiskers plots display values of two different donors. **(F)** CSPG4-CAR T cells, either co-expressing 4-1BB_4-1BB-co-stimulated CCR (green bars) or CD28_4-1BB-co-stimulated CCR (blue bars), were co-cultured with CSPG4^+^ Mel526 and CD20^+^ JeKo-1^WT^ (trans) or CD20^+^ CSPG4^+^ Mel526^CD20^ (cis). Shown are mean and individual values of two different donors (± SD).

Supplementary Figure 2 Proliferative capacity and cytotoxicity of CSPG4-CAR_CD20-CCR T cells upon co-culture with CSPG low-expressing target cells. **(A)** CSPG4 MFI of SupT1 (negative control), A-431 (CSPG4^low^) and Mel526^WT^ (CSPG4^high^) cells. **(B)** Proliferation of CSPG4-CAR_CD20-CCR T cells upon 5 days of co-culture with CSPG4^low^ A-431 or CSPG4^high^ Mel526 target cells, both with or without CCR-stimulating CD20^+^ JeKo-1 cells at an E:T ratio of 1:1. Shown are mean values of two donors (± SD), normalized to proliferation of non-stimulated effector cells. **(C)** CSPG4-CAR_CD20-CCR T cells co-cultured with either A-431 (CSPG4^low^) or Mel526 (CSPG4^high^) cells (in presence of CD20^+^ JeKo-1 cells). Shown are mean values of two donors (± SD) and x-fold change of target cell lysis after 24 hours normalized to start of co-culture.

Supplementary Figure 3 Mel526^WT^ target cell lysis upon co-culture with CSPG4-CAR_CD20-CCR T cells in presence of JeKo-1^WT^ or JeKo-1^CD20^ cells at an E:T ratio of 5:1. Data shows mean values of two donors. Integrated intensity of GFP^+^ Mel526^WT^ target cells was determined over the course of 64 hours of co-culture.
