# Supplementary figures and images for: Combining CSPG4-CAR and CD20-CCR for treatment of metastatic melanoma
Source: Front Immunol. 2023 Oct 11;14:1178060. doi: 10.3389/fimmu.2023.1178060 (PMC10603253; doi:10.3389/fimmu.2023.1178060)

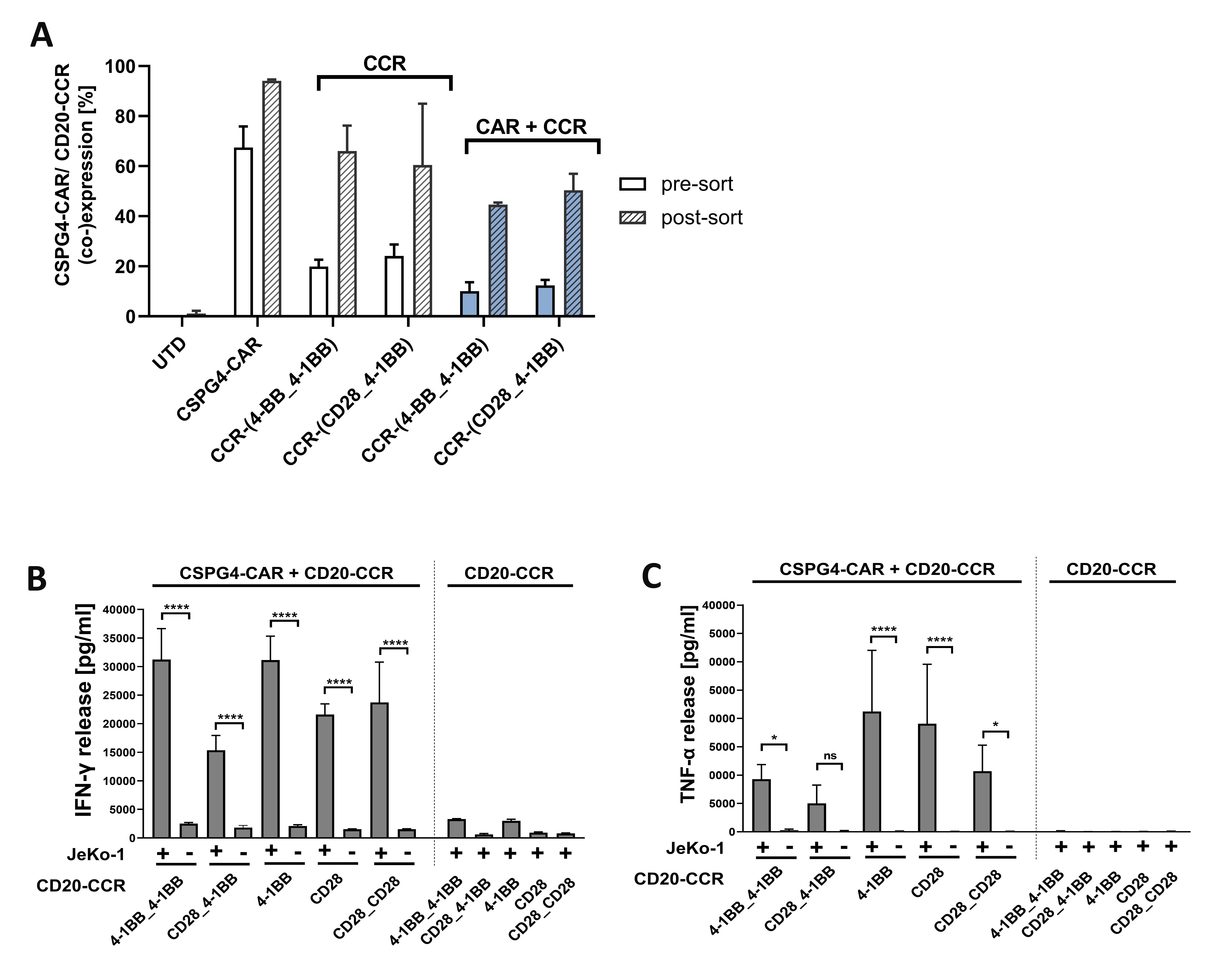

Supplement: Supplementary Figure 1 — Co-expression of CSPG4-CAR and CD20-CCRs, containing various co-stimulatory domains, significantly increases cytokine release upon trans activation. (A) T cells were lentivirally transduced with a CSPG4-CAR, CD20-CCRs (different variants with alternative endodomains displayed in parenthesis) or a combination of both CSPG4-CAR and CD20-CCR (blue bars). The level of (co-)expression is shown pre- and post-enrichment. CAR and CCR expression was determined via co-expression of surface markers ΔLNGFR and ΔEGFR, respectively. Exemplarily shown are mean and individual values of two different donors (± SD). (B, C) Dual-specific T cells co-expressing CSPG4-CAR and CD20-CCR and T cells only expressing CD20-CCR were co-cultured with CSPG4+ Mel526 with our without CD20+ JeKo-1wt cells. Displayed is the IFN-γ secretion after 24 hours of co-culture. Significance was determined using ordinary one-way ANOVA (ns, not significant; *ρ ≤ 0.05, **ρ ≤ 0.01, ***ρ ≤ 0.001, ****ρ ≤ 0.0001). Shown are mean and individual values of two different donors (± SD). (D, E) Dual-specific T cells co-expressing CSPG4-CAR and CD20-CCR and T cells only expressing CD20-CCR or CSPG4-CAR were co-cultured with CSPG4+ Mel526 with our without CD20+ JeKo-1wt cells. Displayed is the TNF-α secretion after 24 hours of co-culture. Box and whiskers plots display values of two different donors. (F) CSPG4-CAR T cells, either co-expressing 4-1BB_4-1BB-co-stimulated CCR (green bars) or CD28_4-1BB-co-stimulated CCR (blue bars), were co-cultured with CSPG4+ Mel526 and CD20+ JeKo-1WT (trans) or CD20+ CSPG4+ Mel526CD20 (cis). Shown are mean and individual values of two different donors (± SD). [file Image_1.jpeg]

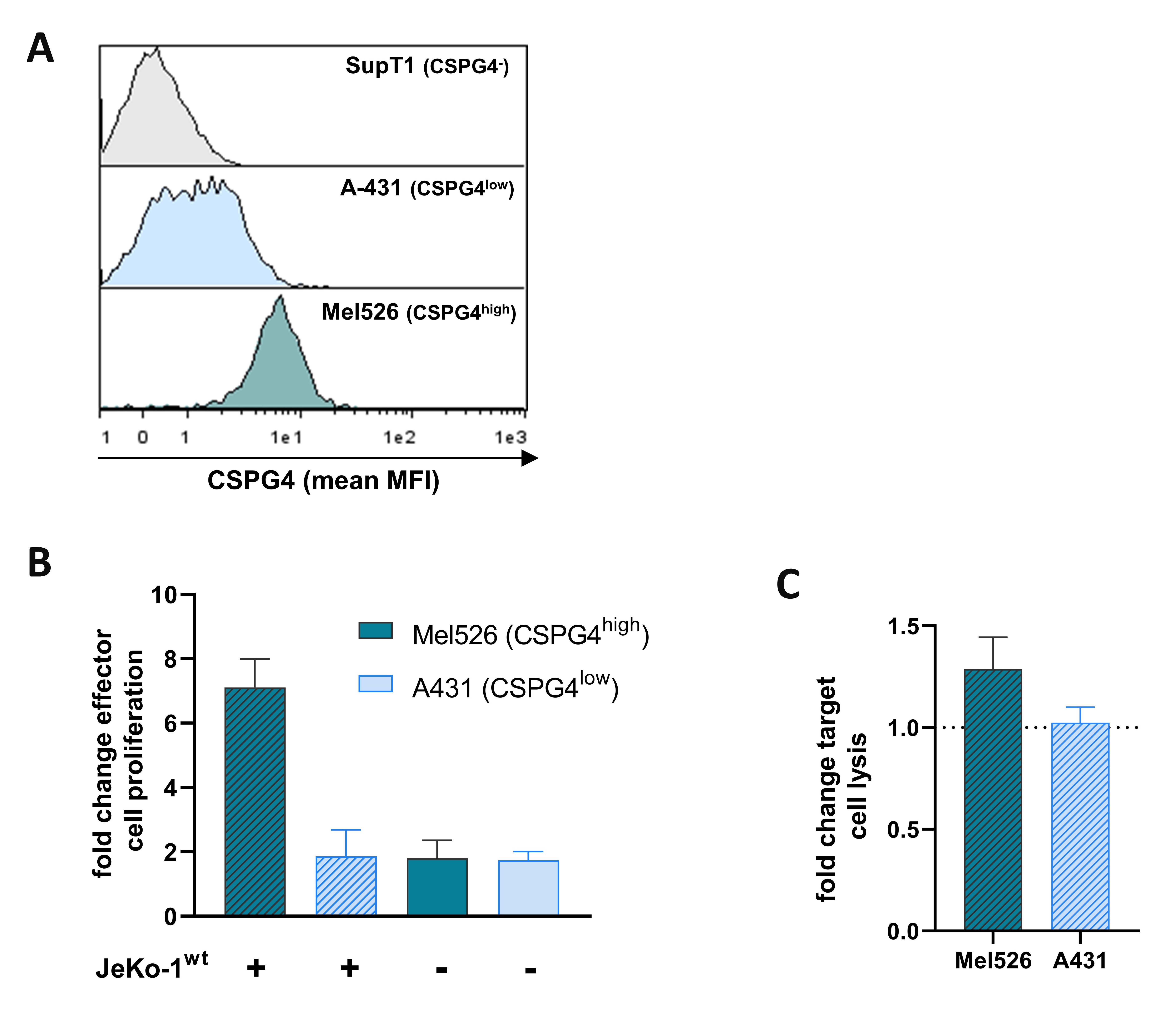

Supplement: Supplementary Figure 2 — Proliferative capacity and cytotoxicity of CSPG4-CAR_CD20-CCR T cells upon co-culture with CSPG low-expressing target cells. (A) CSPG4 MFI of SupT1 (negative control), A-431 (CSPG4low) and Mel526WT (CSPG4high) cells. (B) Proliferation of CSPG4-CAR_CD20-CCR T cells upon 5 days of co-culture with CSPG4low A-431 or CSPG4high Mel526 target cells, both with or without CCR-stimulating CD20+ JeKo-1 cells at an E:T ratio of 1:1. Shown are mean values of two donors (± SD), normalized to proliferation of non-stimulated effector cells. (C) CSPG4-CAR_CD20-CCR T cells co-cultured with either A-431 (CSPG4low) or Mel526 (CSPG4high) cells (in presence of CD20+ JeKo-1 cells). Shown are mean values of two donors (± SD) and x-fold change of target cell lysis after 24 hours normalized to start of co-culture. [file Image_2.jpeg]

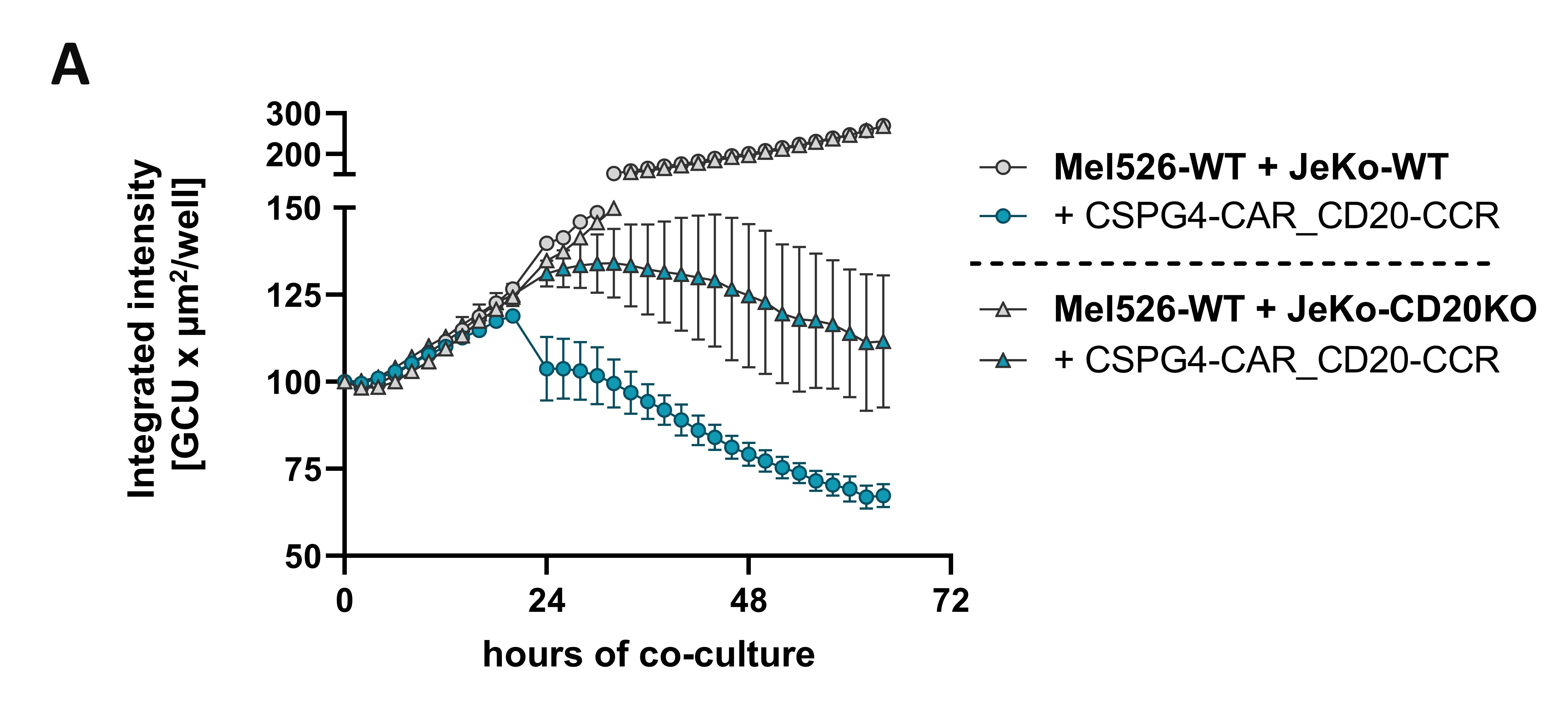

Supplement: Supplementary Figure 3 — Mel526WT target cell lysis upon co-culture with CSPG4-CAR_CD20-CCR T cells in presence of JeKo-1WT or JeKo-1CD20 cells at an E:T ratio of 5:1. Data shows mean values of two donors. Integrated intensity of GFP+ Mel526WT target cells was determined over the course of 64 hours of co-culture. [file Image_3.jpeg]
